# Association of *CELF2* polymorphism and the prognosis of nasopharyngeal carcinoma in southern Chinese population

**DOI:** 10.18632/oncotarget.4870

**Published:** 2015-08-11

**Authors:** Yun-Miao Guo, Ming-Xia Sun, Jing Li, Tong-Tong Liu, Hang-Zhen Huang, Jie-Rong Chen, Wen-Sheng Liu, Qi-Sheng Feng, Li-Zhen Chen, Jin-Xin Bei, Yi-Xin Zeng

**Affiliations:** ^1^ Sun Yat-Sen University Cancer Center, State Key Laboratory of Oncology in South China, Collaborative Innovation Center for Cancer Medicine, Guangzhou, P. R. China; ^2^ Department of Experimental Research, Sun Yat-sen University Cancer Center, Guangzhou, P. R. China; ^3^ Department of Oncology, The First Affiliated Hospital of Zhengzhou University, Zhengzhou, P. R. China; ^4^ Peking Union Medical College, Beijing, P. R. China

**Keywords:** nasopharyngeal carcinoma, SNP, CELF2, prognosis

## Abstract

Nasopharyngeal carcinoma (NPC) is a malignancy with high metastatic potential and loco-regional recurrence. The overall survival of NPC has been limited from further improvement partly due to the lack of effective biomarker for accurate prognosis prediction and precise treatments. Here, in light of the implication of *CELF* gene family in cancer prognosis, we selected 112 tagging single nucleotide polymorphisms (SNPs) located in six members of the family and tested their associations with the clinical outcomes in a discovery cohort of 717 NPC patients. Survival analyses under multivariate cox proportional hazards model and Kaplan–Meier curve revealed five promising SNPs, which were further validated in another independent sample of 1,520 cases. Combined analysis revealed that SNP rs3740194 in *CELF2* was significantly associated with the decreased risk of death with a Hazard ratio (HR) of 0.69 (95% confidence interval [CI] = 0.58–0.82, codominant model). Moreover, rs3740194 also showed a significant association with superior metastasis-free survival (HR = 0.69, 95% CI = 0.57–0.83, codominant model). Taken together, our findings suggested that genetic variant of rs3740194 in *CELF2* gene might be a valuable predictor for NPC prognosis, and potentially useful in the personalized treatment of NPC.

## INTRODUCTION

Nasopharyngeal carcinoma (NPC) is an Epstein-Barr virus (EBV) associated malignant tumor that arises from the epithelial cells at the nasopharynx [1]. It has remarkably high prevalence in southern China and Southeast Asia with an incidence rate of 20–30 per 100, 000, although it's a rare cancer in the western countries [2, 3]. As the tumor cells are sensitive to radiotherapy, substantial improvements in radiation technique and concurrent-adjuvant chemotherapy during the recent decades have provided a significant benefit in clinical outcomes for NPC patients [4]. The 5-year overall survival rate were recently reported to exceed 80% [5–7]. However, there are still 20% to 30% of patients develop distant metastasis and/or loco-regional recurrence, which are the major causes of therapeutic failure [8]. No effective biomarkers other than TNM stage and primary tumor volume are currently used to predict treatment outcomes for NPC patients [9]. However, NPC patients with the same clinical stage usually suffer different clinical outcomes, suggesting the current TNM staging and the tumor size are insufficient factors for prognosis prediction. Therefore, it is urgent to identify effective prognostic biomarkers to improve clinical management for NPC patients. Accumulating evidences have suggested a strong association between germline polymorphisms and cancer prognosis [10]. Previously, genetic polymorphisms in genes *MCP-1* and *HLA-G* have been found to be associated with NPC prognosis [11, 12].

Posttranscriptional regulation of gene expression is a crucial biological event in cancer development [13]. The metabolism of mRNA is largely defined by RNA-binding proteins (RBPs), which play key roles in regulating gene expression at different processes and in cancer progression [14]. The CELF (CUG-BP- and ETR-3-like factor) family of RBPs consist of six members that have been shown with abilities to regulate mRNA editing, stability, and translation [15]. CELF1 and CELF2 [16, 17] are two founder members of CELF family and have been implicated in cell growth, apoptosis, and prognosis of cancer [18–23]. However, the association between CELF family members and the prognosis of NPC remains unclear.

In present study, in attempt to address the link between the genetic variants in members of CELF family and the clinical outcomes of NPC patients, we selected 112 tagging SNPs of *CELF* genes and tested their associations with clinical outcomes in 717 NPC patients, followed by a validation in an additional sample of 1,520 NPC cases.

## RESULTS

### Distribution of patient characteristics and survival status

The characteristics of NPC patients in the discovery stage and the validation stage were summarized in Table 1. The median age at the time of diagnosis was 50 years (ranging 6–98 years) for all the 2,237 patients. Among these patients, there were 1,652 males (73.8%) and 585 females (26.2%). The majority of patients including 1,793 individuals (80.2%) were diagnosed at late stages (III and IV), and the other 444 patients (19.8%) were at early stages (I and II). All of the patients were treated with radiotherapy, including 1,713 patients received 2-dimensional conventional radiotherapy and 524 patients received 3-dimensional radiotherapy. Moreover, different regimes of platinum-based chemotherapy were given to 1,604 patients. Among these patients, 970 patients received inducing chemotherapy, 1,039 patients received concurrent chemotherapy, and 94 patients received adjuvant chemotherapy.

**Table 1:** Characteristic of individuals with NPC in the discovery, validation and combined stages

Characteristics	Discovery stage (*n* = 717)	Validation stage (*n* = 1, 520)	Combined samples (*n* = 2, 237)
	N (%)	N (%)	N (%)
Gender			
Male	525 (73.2)	1127 (74.1)	1652 (73.8)
Female	192 (26.8)	393 (25.9)	585 (26.2)
Age, years			
<50	295 (41.1)	750 (49.3)	1045 (46.7)
≥50	422 (58.9)	770 (50.7)	1192 (53.3)
T-classification			
T1-T2	256 (35.7)	493 (32.4)	749 (33.5)
T3-T4	461 (64.3)	1027 (67.6)	1488 (66.5)
N-classification			
N1-N2	378 (52.7)	822 (54.1)	1200 (53.6)
N3-N4	339 (47.3)	698 (45.9)	1037 (46.4)
Overall stage			
I-II	149 (20.8)	295 (19.4)	444 (19.8)
III-IV	568 (79.2)	1225 (80.6)	1793 (80.2)
Radiotherapy			
2D-RT	614 (85.6)	1099 (72.3)	1713 (76.6)
3D-RT	103 (14.4)	421 (27.7)	524 (23.4)
Chemotherapy			
No	236 (32.9)	397 (22.1)	633 (28.3)
ICT	315 (43.9)	655 (43.1)	970 (43.4)
CCT	279 (38.9)	760 (50.0)	1039 (46.4)
ACT	41 (5.72)	53 (3.49)	94 (4.20)
Death	146 (20.4)	186 (12.2)	332 (14.8)
Metastasis	96 (13.4)	188 (12.4)	284 (12.7)
Recurrence	76 (10.6)	138 (9.08)	214 (9.57)

N, number of patients; 2D-RT, 2-dimensional radiotherapy; 3D-RT, 3-dimensional radiotherapy; ICT, inducing chemotherapy; CCT, concurrent chemotherapy; ACT, adjuvant chemotherapy.

By the last follow-up in June 2013, the median follow-up duration was 56.41 months (Ranging 1.22–119.2 months); 332 patients (14.8%) died of NPC; 284 patients (12.7%) developed distant metastases; 214 patients (9.6%) developed loco-regional recurrences; 26 patients (1.2%) developed both distant metastases and loco-regional recurrences; and, six patients were treated as defaulters due to death of other causes.

### Two-stage association study of *CELF* polymorphisms and NPC survival

After quality control filtering, 112 SNPs in 717 NPC cases were tested in discovery stage by using Cox proportional hazards model adjusted for gender, age, tumor stage, and treatment modality (Table 2 and Supplementary Table S1). Our results showed that five SNPs were significantly associated with NPC survival, including rs3740194 in *CELF2* (HR = 0.69, 95%CI = 0.52–0.90; *P* = 0.007), rs11257025 in *CELF2* (HR = 1.66, 95%CI = 1.18–2.33; *P* = 0.003), rs7094118 in *CELF2* (HR = 1.41, 95%CI = 1.11–1.80; *P* = 0.005), rs7234088 in *CELF4* (HR = 0.64, 95%CI = 0.43–0.95; *P* = 0.029) and rs1786814 in *CELF4* (HR = 0.50, 95%CI = 0.29–0.84; *P* = 0.009), respectively. These five promising SNPs were further genotyped in 1,520 additional cases. Survival analysis revealed that only rs3740194 showed consistently significant association in the validation stage (HR = 0.70, 95%CI = 0.55–0.89, *P* = 0.003), while the other 4 SNPs failed to be validated (*P* > 0.05). Moreover, combined analysis showed that rs3740194 at *CELF2* was significantly associated with the length of overall survival (HR = 0.69, 95%CI = 0.58–0.82, *P* = 4.16 × 10^−5^, *P* corrected for multiple testing = 0.005; Table 2).

**Table 2:** Association results for five candidate SNPs with NPC survival in the discovery, validation and combined stages

SNP	Gene	Alleles^a^	Stages	MAF	GENO^b^	HWE	HR (95%CI)^c^	*P* value	FDR^d^
rs3740194	*CELF2*	G/A							
			Discovery	0.312	61/324/331	0.14	0.69 (0.52-0.90)	0.007	
			Validation	0.325	148/690/679	0.16	0.70 (0.55-0.89)	0.003	
			Combined	0.321	209/1014/1010	0.05	0.69 (0.58-0.82)	4.16 × 10^−5^	0.005
rs11257025	*CELF2*	A/G							
			Discovery	0.101	5/134/576	0.53	1.66 (1.18-2.33)	0.003	
			Validation	0.112	14/312/1192	0.24	1.12 (0.82-1.53)	0.470	
			Combined	0.108	19/446/1768	0.13	1.31 (1.04-1.64)	0.020	0.747
rs7094118	*CELF2*	A/G							
			Discovery	0.295	60/301/353	0.79	1.41 (1.11-1.80)	0.005	
			Validation	0.299	132/644/743	0.67	0.87 (0.69-1.09)	0.230	
			Combined	0.298	192/945/1096	0.58	1.08 (0.91-1.27)	0.383	1.000
rs7234088	*CELF4*	G/A							
			Discovery	0.139	8/182/525	0.08	0.64 (0.43-0.95)	0.029	
			Validation	0.123	22/329/1167	0.91	0.75 (0.54-1.04)	0.087	
			Combined	0.128	30/511/1692	0.25	0.71 (0.55-0.91)	0.008	0.448
rs1786814	*CELF4*	A/G							
			Discovery	0.086	4/115/597	0.81	0.50 (0.29-0.84)	0.009	
			Validation	0.070	7/200/1311	1.00	1.12 (0.76-1.64)	0.583	
			Combined	0.075	11/315/1908	0.76	0.80 (0.59-1.10)	0.170	1.000

a Minor allele/major allele.

b Minor homozygote/heterozygote/major homozygote.

c Under codominant model.

d Benjamini-Hochberg correction for multiple testing. MAF, minor allele frequency; HWE, Hardy-Weinberg equilibrium; HR, Hazard Ratio, adjusted for gender, age, tumor stage, and treatment; CI, confidence interval.

### Association analyses of *CELF2* rs3740194 as a prognostic factor of NPC

**Figure 1:**
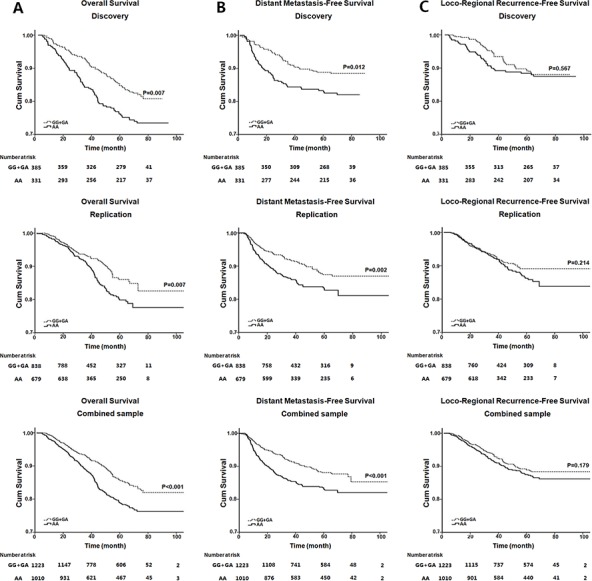
Kaplan–Meier survival analysis of rs3740194 in the discovery, validation and combined samples **A.** Kaplan–Meier plots of overall survival in NPC patients; **B.** Kaplan–Meier plots of distant metastasis-free survival in NPC patients; **C.** Kaplan–Meier plots of loco-regional recurrence-free survival in NPC patients. *P* values were derived from log-rank tests.

According to the univariate analyses, both the risk of death and metastasis were significantly increased in males, patients with advanced T, N and overall stages, and patients treated with inducing chemotherapy (Table 3). Moreover, elder age was an adverse factor for overall survival (HR = 1.83, 95%CI = 1.46–2.31). None of the selected factors was associated with recurrence, except that being male or receiving 3-dimensional radiotherapy was associated with poor loco-regional recurrence-free survival, respectively (Table 3). Consistent with the above Cox proportional hazards model, rs3740194 at *CELF2* showed a significant association with overall survival (AG+GG vs AA, HR = 0.66, 95% CI = 0.53–0.82). Moreover, it was significantly associated with the metastasis-free survival (AG+GG vs AA, HR = 0.63, 95% CI = 0.50–0.80). The Kaplan-Meier survival curves and log-Rank tests showed that the AA genotype of rs3740194 was consistently associated with poor overall survival and metastasis-free survival of NPC patients in the discovery, validation and combined samples (*P* < 0.05; Figure 1).

**Table 3:** Univariate analysis of prognostic factors associated with NPC prognosis

Variables	OS			DMFS			LRRFS		
	Deaths (%)	HR (95% CI)	*P* value	Metastases (%)	HR (95% CI)	*P* value	Recurrences (%)	HR (95% CI)	*P* value
Gender									
Female	55 (9.40)	1	5.97 × 10^−6^	53 (9.06)	1	0.001	41 (7.01)	1	0.005
Male	277 (16.8)	1.95 (1.46–2.61)		231 (14.0)	1.65 (1.22–2.22)		173 (10.5)	1.63 (1.16–2.30)	
Age (years)									
<50	105 (10.0)	1	2.77 × 10^−7^	122 (11.7)	1	0.262	100 (9.57)	1	0.833
≥50	227 (19.0)	1.83 (1.46–2.31)		162 (13.6)	1.14 (0.90–1.45)		114 (9.56)	0.97 (0.74–1.27)	
T status									
T1-T2	71 (9.48)	1	3.86 × 10^−7^	73 (9.75)	1	0.002	77 (10.3)	1	0.636
T3-T4	261 (17.5)	1.97 (1.52–2.57)		211 (14.2)	1.52 (1.16–1.98)		137 (9.21)	0.93 (0.71–1.24)	
N status									
N1-N2	138 (11.5)	1	2.84 × 10^−7^	118 (9.83)	1	3.28 × 10^−6^	110 (9.17)	1	0.208
N3-N4	194 (18.7)	1.77 (1.42–2.20)		166 (16.0)	1.75 (1.38–2.22)		104 (10.0)	1.19 (0.91-1.55)	
Overall stage									
I-II	31 (6.98)	1	1.95 × 10^−7^	30 (6.76)	1	2.15 × 10^−5^	45 (10.1)	1	0.949
III-IV	301 (16.8)	2.67 (1.84–3.86)		254 (14.2)	2.27 (1.56–3.32)		169 (9.43)	1.01 (0.73–1.40)	
Radiotherapy									
2D-RT	272 (15.9)	1	0.203	231 (13.5)	1	0.147	157 (9.17)	1	0.042
3D-RT	60 (11.5)	0.83 (0.63–1.10)		53 (10.1)	0.80 (0.59–1.08)		57 (10.9)	1.37 (1.01–1.86)	
Chemotherapy									
No	85 (13.4)	1		55 (8.69)	1		71 (11.2)	1	0.326
ICT	166 (17.1)	1.41 (1.14–1.75)	0.002	142 (14.6)	1.38 (1.09–1.74)	0.007	96 (9.90)	1.15 (0.87–1.50)	
CCT	140 (13.5)	0.96 (0.77–1.19)	0.712	132 (12.7)	1.07 (0.85–1.35)	0.564	93 (8.95)	0.99 (0.75–1.29)	0.913
ACT	20 (21.3)	1.49 (0.95–2.35)	0.083	16 (17.0)	1.39 (0.84–2.30)	0.201	8 (8.51)	0.90 (0.44–1.82)	0.764
*CELF2* rs3740194 genotype									
AA	181 (17.9)	1		158 (15.6)	1		105 (8.91)	1	
AG	132 (13.0)	0.70 (0.56–0.87)	0.002	107 (10.6)	0.65 (0.51–0.83)	5.05 × 10^−4^	90 (8.88)	0.82 (0.62–1.09)	0.164
GG	19 (9.09)	0.70 (0.55–0.89)	0.003	19 (9.09)	0.75 (0.59–0.95)	0.018	19 (9.09)	0.93 (0.73–1.18)	0.538
AG+GG	151 (12.3)	0.66 (0.53–0.82)	1.78 × 10^−4^	126 (10.3)	0.63 (0.50–0.80)	1.21 × 10^−4^	109 (8.91)	0.82 (0.63–1.08)	0.159

OS, Overall Survival; DMFS, Distant Metastasis-Free Survival; LRRFS, Loco-Regional Recurrence-Free Survival; 2D-RT, 2-dimensional radiotherapy; 3D-RT, 3-dimensional radiotherapy; ICT, inducing chemotherapy; CCT, concurrent chemotherapy; ACT, adjuvant chemotherapy; HR, Hazard Ratio, derived from COX proportional hazards model; CI, confidence interval.

The multivariate analysis revealed that the rs3740194 AA genotype was a significant independent predictor for the inferior overall survival (AA vs AG+GG, HR = 1.53, 95%CI = 1.23–1.89) and metastasis-free survival (AA vs AG+GG, HR = 1.60, 95%CI = 1.26–2.02). Moreover, gender was shown as an independent factor for all prognostic measures (*P* < 0.05, Table 4). The younger age, early T and N stages were also independent indicators for superior overall survival, whereas the advanced T and N stage were independent factors for inferior metastasis-free survival (*P* < 0.05, Table 4). In addition, no significant association was observed between rs3740194 genotypes and any of the selected clinical characteristics (*P* > 0.05, Supplementary Table S2).

**Table 4:** Multivariate analysis of prognostic factors associated with NPC prognosis

Variables	OS		DMFS		LRRFS	
	HR (95% CI)	*P* value	HR (95% CI)	*P* value	HR (95% CI)	*P* value
Gender (male vs. female)	1.95 (1.45–2.61)	7.68 × 10^−6^	1.65 (1.22–2.23)	0.001	1.61 (1.14–2.27)	0.007
Age (≥ vs. < 50 years)	1.85 (1.47–2.34)	2.33 × 10^−7^	1.15 (0.90–1.45)	0.262	0.98 (0.75–1.29)	0.906
T status (T3-T4 vs. T1-T2)	1.88 (1.43–2.47)	6.12 × 10^−6^	1.40 (1.06–1.84)	0.018	0.92 (0.68–1.23)	0.560
N status (N2-N3 vs. N0-N1)	1.77 (1.41–2.21)	8.68 × 10^−7^	1.66 (1.30–2.12)	4.99 × 10^−5^	1.22 (0.92–1.62)	0.162
Radiotherapy (3D-RT vs. 2D-RT)	0.94 (0.70–1.27)	0.691	0.82 (0.60–1.12)	0.206	1.39 (1.01–1.92)	0.045
Inducing chemotherapy (Yes vs. No)	1.04 (0.82–1.30)	0.758	1.11 (0.87–1.43)	0.404	1.08 (0.81–1.45)	0.590
Concurrent chemotherapy (Yes vs. No)	0.85 (0.67–1.08)	0.177	1.00 (0.77–1.29)	0.990	0.89 (0.67–1.20)	0.459
Adjuvant chemotherapy (Yes vs. No)	1.36 (0.86–2.17)	0.192	1.24 (0.74–2.07)	0.416	0.89 (0.43–1.82)	0.744
*CELF2* rs3740194 (AA vs AG)	1.44 (1.15–0.81)	0.001	1.55 (1.21–1.99)	4.45 × 10^−4^	1.22 (0.92–1.62)	0.167
*CELF2* rs3740194 (AA vs GG)	1.46 (1.15–1.86)	1.21 × 10^−4^	1.36 (1.07–1.73)	0.012	1.12 (0.87–1.43)	0.374
*CELF2* rs3740194 (AA vs AG+GG)	1.53 (1.23–1.89)	1.30 × 10^−4^	1.60 (1.26–2.02)	8.87 × 10^−5^	1.21 (0.93–1.59)	0.156

OS, Overall Survival; DMFS, Distant Metastasis-Free Survival; LRRFS, Loco-Regional Recurrence-Free Survival; 2D-RT, 2-dimensional radiotherapy; 3D-RT, 3-dimensional radiotherapy; HR, Hazard Ratio, derived from COX proportional hazards model; CI, confidence interval.

Stratified analysis was also conducted to estimate the effect of different treatment modalities on the association between rs3740194 genotypes and NPC prognosis. The most significant associations were found between OS and the patients treated with 2D-RT only (HR = 1.88, 95%CI = 1.27–2.81; *P* = 0.002; Table 5) and those treated with 2D-RT plus CCT (HR = 1.60, 95% CI = 1.13–2.26; *P* = 0.007; Table 5). However, no significant differences were observed among subgroups (*P* > 0.05 for heterogeneity test), suggesting that the effect of different treatment regimes on the association of rs3740194 and NPC prognosis is minimal.

**Table 5:** Analysis on the association between rs3740194 genotype and NPC prognosis stratified by treatment modality

RT	CRT	rs3740194			OS		DMFS		LRRFS	
		AA (%)	AG (%)	GG (%)	HR (95% CI)^a^	*P* value	HR (95% CI)^a^	*P* value	HR (95% CI)^a^	*P* value
2D	No	235 (45.1)	240 (46.1)	46 (8.80)	1.88 (1.27–2.81)	0.002	1.91 (1.16–3.17)	0.012	1.07 (0.69–1.65)	0.761
2D	ICT	329 (43.7)	342 (45.4)	82 (10.9)	1.17 (0.90–1.51)	0.247	1.26 (0.95–1.67)	0.105	1.40 (0.96–2.04)	0.078
2D	CCT	300 (44.8)	308 (46.0)	62 (9.20)	1.60 (1.13–2.26)	0.007	1.39 (0.99–1.96)	0.056	1.24 (0.83–1.85)	0.296
2D	ACT	36 (47.4)	38 (50.0)	2 (2.60)	1.60 (0.65–3.93)	0.310	1.64 (0.61–4.41)	0.329	0.55 (0.12–2.59)	0.446
3D	No	54 (48.2)	49 (43.8)	9 (8.00)	1.58 (0.41–6.07)	0.502	1.34 (0.35–5.09)	0.670	1.24 (0.56–2.72)	0.596
3D	ICT	99 (45.6)	97 (44.7)	21 (9.70)	1.67 (0.90–3.12)	0.104	1.92 (0.90–4.10)	0.092	0.91 (0.53–1.58)	0.748
3D	CCT	178 (48.2)	159 (43.1)	32 (8.70)	1.52 (0.95–2.46)	0.084	1.77 (1.04–3.01)	0.035	0.89 (0.52–1.53)	0.681
3D	ACT	9 (50.0)	8 (44.4)	1 (5.60)	N/A	0.999	N/A	1.000	N/A	1.000
*P*_heterogeneity_						0.660		0.854		0.811

a Codominant model; adjusted for gender, age, and tumor stage. OS, Overall Survival; DMFS, Distant Metastasis-Free Survival; LRRFS, Loco-Regional Recurrence-Free Survival; RT, Radiotherapy; CRT, Chemoradiotherapy; 2D, 2-dimensional; 3D, 3-dimensional; ICT, inducing chemotherapy; CCT, concurrent chemotherapy; ACT, adjuvant chemotherapy; HR, Hazard Ratio; CI, confidence interval.

## DISCUSSION

Although NPC tumor cells are sensitive to chemo/radiotherapy, a proportion of NPC patients, especially those at later stages, develop distant metastasis and loco-regional recurrence, leading to poor outcomes. Identification of effective biomarkers for treatment optimizations is important to improve the NPC prognosis. Herein, we carried out a two-stage survival analysis with large sample size and explored the association of polymorphisms of *CELF* genes with NPC outcome. To the best of our knowledge, this is the first study to demonstrate associations of genetic variations in *CELF* genes or RBPs with NPC outcome. By combining a total of 2, 237 cases, we found that rs3740194 at *CELF2* locus was significantly associated with NPC overall survival rate and metastasis-free survival rate (both *P* < 0.001), and further consistently, rs3740194 at *CELF2* locus was an independent prognostic factor for overall survival and metastasis-free survival. These suggest that the CELF2 might play important roles in the metastasis and invasion of NPC, which have been shown as the major causes of its poor survival [8].

Previous studies have reported that RBPs are involved in cancer by modulating cell growth and proliferation [24]. As a RBP, CELF2 (also known as CUGBP2, ETR3, BRUNOL2, Napor2) is a family member of CELF that was identified as a transcript highly expressed in neuroblastoma cells undergoing colchicine-induced apoptosis [25, 26]. CELF2 is ubiquitously expressed, while overexpression of CELF2 leads to mitotic catastrophe and apoptosis of cells [21, 27, 28]. In colon cancer cells, CELF2 expression is consistently reduced during neoplastic transformation, and suppression of CELF2 expression decreased radiation-induced apoptosis, suggesting it is a potential tumor suppressor protein [22, 29]. Supportively, overexpression of CELF2 resulted in mitotic catastrophe of pancreatic cancer cells [18]. CELF2 shares similar structure with HuR, which is one of the well-known RBPs that play important roles in tumor development [30, 31]. CELF2 can bind to the ARE sequence in the 3′-UTR of cyclooxygenase-2 (COX-2), increase COX-2 mRNA stability, and however, inhibit the translation of COX-2. By contrast, HuR specifically interacted with the AU-rich element (ARE) of COX-2, leading to increased expression of COX-2 [32]. The expression of cytoplasmic HuR was significantly associated with COX-2 expression in tumors and showed a correlation with lymphatic invasion and metastasis [33, 34]. Furthermore, CELF2 competed with HuR for binding to COX-2 mRNA and prevented its translation [35].

To further investigate the functional role of rs3740194, which is located on the fourth intron of *CELF2*, we conducted eQTL analyses in human lymphoblastoid cell lines of 45 unrelated Han Chinese individuals. Gene expression data were obtained from the NCBI Gene Expression Omnibus (series number: GSE6536; http://www.ncbi.nlm.nih.gov/geo/), and SNP genotype data were derived from the corresponding HapMap dataset (Rel28, PhaseII+III, August 10, Build 36; http://hapmap.ncbi.nlm.nih.gov/). The eQTL effects were classified as *cis* or *trans*, if they occurred within an expanded region (±1 Mb) surrounding rs3740194, or outside this region, respectively. However, none of significant *cis*-eQTLs or *trans*-eQTLs was observed after Benjamini-Hochberg correction for multiple testing (*P* < 0.05, data not shown). These results might be limited by the single cell line data and incomplete coverage of the probe sets at this region. Further investigations are warranted to reveal the functional relevance of the SNP association.

Taken together, we carried out a two-stage survival analysis to address the associations between the polymorphisms of *CELF* family members and NPC prognosis, and discovered that NPC patients with AA genotype of *CELF2* gene polymorphism rs3740194 correlated with the inferior overall survival and metastasis-free survival. The finding suggests that CELF2 might be another potential prognostic predictor of NPC, which could be applied for stratified therapeutic studies attempting to develop better treatment and better outcome for NPC. In addition, we acknowledged that our study is limited as a retrospective study in a single center. Further studies in either prospective manner or multicenter may help to validate our findings.

## MATERIALS AND METHODS

### Subject recruitment

All subjects were histologically diagnosed with NPC and subsequently treated at Sun Yat-sen University Cancer Center (SYSUCC) between January 2002 and December 2010. Individuals were excluded if they reported with history of cancer, and radiotherapy or chemotherapy at diagnosis. A total of 2, 237 NPC patients were enrolled, in which 717 patients were randomly selected into the discovery stage and the remaining 1, 520 patients were treated as validation sample. All subjects were staged according to the sixth edition of UICC/AJCC TNM staging system [36]. The follow-up data was collected every six months after diagnosis or until death. Local recurrence was confirmed by fiberoptic endoscopy, magnetic resonance imaging (MRI) and biopsy, whereas distant metastases were diagnosed by clinical symptoms, physical examination, and imaging methods including CT-scan, bone scan, abdominal sonography or 18F-fluorodeoxyglucose positron emission tomography and computed tomography (PET-CT). This study was approved by the ethics committees of SYSUCC. Informed consent documents were obtained from all of the subjects.

### SNP selection and genotyping

A total of 734 SNPs located in the coding regions and untranslated-regions (5′-UTR and 3′-UTR) of six CELF members were retrieved from the International HapMap database (Rel28, PhaseII+III, August 10, Build 36; http://hapmap.ncbi.nlm.nih.gov/) and the NCBI database (dbSNP version 126, http://www.ncbi.nlm.nih.gov), if their minor allele frequencies (MAF) in Chinese Han population (CHB) were above 5%. Subsequently, 114 tagging SNPs were identified by linkage disequilibrium (LD) analysis using Haploview software (version 4.2; *r*^2^ < 0.5).

Venous blood sample was collected from each patient prior to any treatment. DNA was extracted from blood samples using the QIAamp DNA Blood Midi Kit (QIAGEN, Valencia, CA). In discovery stage, candidate SNPs were genotyped in 717 NPC patients by using GoldenGate^®^ Genotyping Assay (Illumina Inc, San Diego, CA) according to manufacturer's instructions. Two SNPs were excluded due to genotyping failure (call rates < 95%). The overall call rates ranged from 98.7% to 100% for the remaining 112 SNPs. Five promising SNPs with significant *P*-value (<0.05) in discovery stage were further genotyped in the validation sample of 1, 520 NPC patients, using TaqMan assay on ABI PRISM 7900 HT platform (Applied Biosystems Inc.). The details of primers and probes were shown in Supplementary Table S3.

### Statistical analysis

Minor allele frequency was calculated and Hardy–Weinberg equilibrium (HWE) was tested for each selected SNP using PLINK software (version 1.07). The analyses of association between candidate SNPs and length of survival were carried out under different genetic models (Supplementary Table S4). Hazard ratios (HRs) and 95% confidence intervals (95% CIs) were calculated by Cox proportional hazards regression model with adjustments for age, sex, clinical stage, and treatment modality, considering their influence on the length of survival. Comparisons of demographic characteristics, selected variables and genotypes were analyzed by the Wilcoxon rank-sum test (for continuous variables) or χ2 test (for categorical variables). The survival curves were generated by Kaplan-Meier methods, and *P* values were assessed by log-rank tests. The hazard ratios (HRs) and 95% confidence intervals (95% CIs) for univariate analyses were calculated using Cox proportional hazards model. Multivariate survival analyses were adjusted for age, gender, T stage, N stage, and treatment modality. These analyses were carried out in a two-sided manner by using R version 3.0.2.

## SUPPLEMENTARY TABLES


