# Supplementary material for: Association of CELF2 polymorphism and the prognosis of nasopharyngeal carcinoma in southern Chinese population
Source: Oncotarget. 2015 Aug 11;6(29):27176–86. doi: 10.18632/oncotarget.4870 (PMC4694981; doi:10.18632/oncotarget.4870)
Supplement: Supplementary file 1 [file oncotarget-06-27176-s001.pdf]

SUPPLEMENTARY TABLES

Supplementary Table S1: Association results between SNPs in *CELF* genes and overall survival of NPC patients in the discovery stage

SNP	Gene	Chr.	Position	Alleles[a]	MAF	GENO[b]	HWE	HR (95%CI)[c]	*P* value[c]
rs3740194	*CELF2*	10	11297309	G/A	0.312	61/324/331	0.163	0.69 (0.52–0.90)	0.007
rs11257025	*CELF2*	10	11275763	A/G	0.101	5/134/576	0.533	1.66 (1.18–2.33)	0.003
rs7094118	*CELF2*	10	11256442	A/G	0.295	60/301/353	0.787	1.41 (1.11–1.80)	0.005
rs7234088	*CELF4*	18	35001400	G/A	0.139	8/182/525	0.084	0.64 (0.43–0.95)	0.029
rs1786814	*CELF4*	18	35077028	A/G	0.086	4/115/597	0.810	0.50 (0.29–0.84)	0.009
rs1999207	*CELF2*	10	11078390	A/G	0.230	33/263/418	0.343	0.79 (0.60–1.04)	0.096
rs6507207	*CELF4*	18	35134455	G/A	0.132	12/165/537	0.869	0.69 (0.54–1.07)	0.108
rs7537265	*CELF3*	1	151686240	A/C	0.209	32/235/450	0.822	1.28 (0.95–1.74)	0.111
rs932918	*CELF2*	10	11081947	G/A	0.314	66/317/333	0.446	0.83 (0.65–1.07)	0.155
rs10502673	*CELF4*	18	35062163	A/G	0.354	92/322/301	0.683	0.83 (0.66–1.04)	0.101
rs9957642	*CELF4*	18	35074714	G/A	0.152	18/181/515	0.663	0.74 (0.55–1.02)	0.058
rs1624142	*CELF2*	10	11176650	A/G	0.345	86/321/308	0.869	0.85 (0.67–1.08)	0.187
rs11879556	*CELF5*	19	3278206	G/A	0.282	55/294/367	0.782	0.84 (0.65–1.08)	0.180
rs1539848	*CELF4*	18	34981616	A/G	0.373	93/347/275	0.338	1.27 (0.99–1.64)	0.064
rs2653517	*CELF2*	10	11294858	G/A	0.415	129/341/244	0.605	1.24 (0.98–1.57)	0.076
rs11039260	*CELF1*	11	47509700	A/C	0.287	61/289/365	0.716	0.81 (0.63–1.04)	0.093
rs8182392	*CELF4*	18	35027184	G/A	0.172	26/194/497	0.190	0.81 (0.62–1.07)	0.134
rs11257055	*CELF2*	10	11355431	A/G	0.363	90/340/287	0.519	0.82 (0.64–1.03)	0.089
rs2000697	*CELF4*	18	35078723	C/A	0.441	139/358/219	0.735	1.18 (0.93–1.49)	0.172
rs619030	*CELF2*	10	11206281	G/A	0.404	125/329/262	0.215	0.86 (0.69–1.08)	0.188
rs1291836	*CELF2*	10	11101276	A/G	0.226	33/257/425	0.521	0.81 (0.62–1.07)	0.138
rs2279809	*CELF2*	10	11360673	G/A	0.477	160/361/193	0.764	1.22 (0.96–1.54)	0.101
rs679944	*CELF2*	10	11197845	A/G	0.284	62/281/369	0.408	0.85 (0.67–1.09)	0.191
rs2315915	*CELF5*	19	3277094	G/A	0.181	21/217/478	0.615	0.83 (0.62–1.11)	0.207
rs4807434	*CELF5*	19	3259054	A/C	0.177	20/213/483	0.609	0.76 (0.56–1.02)	0.065
rs4750035	*CELF2*	10	11296726	A/G	0.089	3/121/592	0.350	1.34 (0.86–2.09)	0.191
rs2242081	*CELF1*	11	47500267	G/A	0.466	160/341/213	0.291	0.86 (0.68–1.08)	0.186
rs3781053	*CELF2*	10	11359469	A/G	0.373	100/334/281	1.000	1.21 (0.95–1.54)	0.126
rs1058348	*CELF2*	10	11302345	A/G	0.079	5/103/608	0.795	1.38 (0.85–2.22)	0.192
rs12440927	*CELF6*	15	72595888	G/A	0.157	20/185/510	0.483	0.80 (0.59–1.07)	0.136
rs12454639	*CELF4*	18	35078535	A/G	0.073	1/102/612	0.165	1.45 (0.86–2.47)	0.165
rs3814629	*CELF2*	10	11377471	G/A	0.334	68/342/305	0.053	1.21 (0.93–1.57)	0.149
rs2653521	*CELF2*	10	11290351	A/G	0.213	39/227/450	0.148	1.21 (0.91–1.62)	0.188
rs2912221	*CELF6*	15	72602709	G/A	0.077	5/100/612	0.599	0.76 (0.51–1.15)	0.195

(*Continued*)

SNP	Gene	Chr.	Position	Alleles[a]	MAF	GENO[b]	HWE	HR (95%CI)[c]	*P* value[c]
rs17453876	*CELF2*	10	11331406	A/C	0.054	1/ 76/640	0.715	1.36 (0.77–2.40)	0.286
rs1787633	*CELF4*	18	34847052	A/G	0.199	33/219/465	0.291	0.80 (0.61–1.04)	0.098
rs650371	*CELF2*	10	11199814	A/C	0.164	19/197/500	1.000	0.87 (0.65–1.17)	0.360
rs12610909	*CELF5*	19	3252909	A/G	0.365	95/333/289	1.000	1.12 (0.88–1.42)	0.357
rs2246449	*CELF2*	10	11356160	A/G	0.440	143/343/229	0.495	0.88 (0.70–1.10)	0.258
rs12248765	*CELF2*	10	11142727	G/A	0.243	45/258/414	0.611	0.88 (0.67–1.14)	0.328
rs312926	*CELF5*	19	3275315	A/G	0.276	53/288/374	0.852	0.85 (0.66–1.10)	0.215
rs6507196	*CELF4*	18	34874772	A/C	0.491	184/335/197	0.086	0.87 (0.69–1.09)	0.218
rs1786795	*CELF4*	18	35034260	A/C	0.031	0/ 44/673	1.000	0.72 (0.37–1.38)	0.324
rs1941945	*CELF4*	18	35000087	A/G	0.172	14/218/484	0.066	1.19 (0.85–1.67)	0.300
rs6507201	*CELF4*	18	35016217	G/A	0.414	123/347/246	1.000	0.87 (0.69–1.10)	0.240
rs9954961	*CELF4*	18	35116950	A/G	0.242	48/251/417	0.223	1.07 (0.82–1.40)	0.638
rs660676	*CELF2*	10	11215311	G/A	0.370	96/336/282	0.872	0.93 (0.73–1.18)	0.530
rs11256991	*CELF2*	10	11166455	C/A	0.078	5/101/609	0.606	0.89 (0.59–1.35)	0.577
rs1791480	*CELF4*	18	34987926	A/G	0.095	2/132/582	0.051	0.87 (0.59–1.28)	0.469
rs948540	*CELF4*	18	35091451	G/A	0.165	21/194/499	0.684	0.85 (0.63–1.14)	0.277
rs2762537	*CELF2*	10	11110062	G/A	0.106	11/130/574	0.238	0.95 (0.66–1.37)	0.777
rs4366786	*CELF4*	18	34860337	A/C	0.140	15/171/530	0.757	0.89 (0.65–1.23)	0.492
rs4750031	*CELF2*	10	11274572	A/G	0.392	112/338/267	0.755	1.08 (0.85–1.37)	0.523
rs312058	*CELF5*	19	3227102	G/A	0.359	94/325/295	0.746	0.93 (0.73–1.18)	0.542
rs1786809	*CELF4*	18	35093291	A/G	0.031	0/ 44/672	1.000	1.35 (0.66–2.77)	0.406
rs1539850	*CELF4*	18	35078859	A/G	0.028	1/ 38/674	0.430	1.20 (0.53–2.71)	0.657
rs312934	*CELF5*	19	3268722	A/G	0.166	24/190/501	0.280	0.92 (0.68–1.24)	0.576
rs1291818	*CELF2*	10	11132190	A/G	0.192	27/221/467	0.904	0.90 (0.68–1.20)	0.487
rs10905929	*CELF2*	10	11345047	A/G	0.050	1/ 70/645	1.000	1.26 (0.71–2.22)	0.433
rs1910231	*CELF2*	10	11342601	A/G	0.329	78/314/323	0.932	0.94 (0.74–1.20)	0.633
rs9304173	*CELF4*	18	35061197	G/A	0.155	15/192/508	0.668	1.10 (0.79–1.52)	0.580
rs4747895	*CELF2*	10	11237509	A/G	0.378	114/313/288	0.067	0.97 (0.77–1.22)	0.813
rs12570743	*CELF2*	10	11248497	G/A	0.394	118/328/270	0.274	0.96 (0.77–1.21)	0.753
rs658469	*CELF2*	10	11221366	A/G	0.194	30/218/467	0.474	0.94 (0.71–1.25)	0.691
rs1786060	*CELF4*	18	34833046	A/G	0.122	7/161/547	0.227	1.20 (0.82–1.74)	0.347
rs2009759	*CELF4*	18	34954021	A/G	0.327	77/313/325	0.932	1.16 (0.90–1.50)	0.253
rs2378991	*CELF2*	10	11369250	A/G	0.403	117/342/256	0.877	1.10 (0.87–1.39)	0.443
rs1539847	*CELF4*	18	34981800	G/A	0.237	34/272/410	0.216	1.14 (0.85–1.52)	0.376
rs1786767	*CELF4*	18	35094574	G/A	0.450	143/359/215	0.821	0.87 (0.69–1.10)	0.239
rs11876166	*CELF4*	18	34962842	A/G	0.246	37/278/400	0.227	1.08 (0.82–1.42)	0.579
rs1786068	*CELF4*	18	34854121	A/C	0.160	23/183/511	0.209	0.88 (0.65–1.18)	0.388

(*Continued*)

SNP	Gene	Chr.	Position	Alleles[a]	MAF	GENO[b]	HWE	HR (95%CI)[c]	*P* value[c]
rs9888023	*CELF2*	10	11349592	G/A	0.201	30/228/459	0.816	0.92 (0.70–1.23)	0.589
rs2187048	*CELF4*	18	35033025	G/A	0.158	16/195/506	0.675	1.03 (0.75–1.42)	0.848
rs1786783	*CELF4*	18	34969189	G/A	0.054	1/ 75/639	0.715	0.86 (0.53–1.41)	0.551
rs4077472	*CELF4*	18	34893326	A/G	0.250	48/261/406	0.486	1.10 (0.85–1.43)	0.470
rs3780992	*CELF2*	10	11312042	G/A	0.169	15/211/489	0.182	1.09 (0.79–1.51)	0.602
rs2959930	*CELF6*	15	72598614	G/A	0.453	142/366/209	0.452	1.08 (0.85–1.36)	0.538
rs1539849	*CELF4*	18	35074002	A/C	0.116	5/156/555	0.102	1.25 (0.84–1.85)	0.278
rs4799926	*CELF4*	18	35026794	A/G	0.140	9/183/524	0.161	0.86 (0.61–1.21)	0.381
rs11817658	*CELF2*	10	11296839	A/C	0.267	40/301/372	0.044	1.14 (0.86–1.50)	0.373
rs11257046	*CELF2*	10	11327285	G/A	0.301	56/318/341	0.132	1.06 (0.81–1.38)	0.678
rs1875594	*CELF2*	10	11345345	A/G	0.089	3/121/591	0.351	1.07 (0.70–1.63)	0.771
rs2000698	*CELF4*	18	35108655	A/G	0.488	168/361/185	0.822	1.00 (0.80–1.26)	0.969
rs2653514	*CELF2*	10	11298352	A/G	0.083	2/115/598	0.217	1.15 (0.74–1.79)	0.546
rs2027755	*CELF4*	18	35042998	A/G	0.159	17/193/506	0.889	0.94 (0.69–1.28)	0.675
rs7102372	*CELF1*	11	47496827	A/G	0.057	0/ 82/632	0.160	0.92 (0.57–1.48)	0.733
rs1786802	*CELF4*	18	35072150	A/G	0.427	134/343/238	0.593	1.06 (0.84–1.34)	0.620
rs7242303	*CELF4*	18	34964005	A/G	0.425	123/363/230	0.359	0.91 (0.72–1.16)	0.462
rs7899913	*CELF2*	10	11224830	A/G	0.056	3/ 74/640	0.478	0.92 (0.58–1.45)	0.719
rs17750345	*CELF4*	18	35079222	A/C	0.241	42/261/413	0.919	0.98 (0.74–1.29)	0.871
rs1954869	*CELF4*	18	35041896	C/A	0.353	83/339/293	0.327	1.02 (0.80–1.31)	0.870
rs13381492	*CELF4*	18	34882659	G/A	0.086	9/105/601	0.091	1.10 (0.72–1.70)	0.655
rs3889769	*CELF4*	18	34923021	G/A	0.413	133/325/258	0.090	1.01 (0.80–1.28)	0.908
rs2027754	*CELF4*	18	35042882	G/A	0.316	70/311/333	0.863	1.00 (0.78–1.28)	0.999
rs4799942	*CELF4*	18	35117522	A/G	0.216	32/245/439	0.826	1.04 (0.78–1.38)	0.806
rs3897638	*CELF4*	18	34897450	A/G	0.223	35/249/432	1.000	1.07 (0.81–1.42)	0.647
rs2938021	*CELF2*	10	11292972	A/G	0.340	80/328/309	0.678	0.95 (0.75–1.21)	0.681
rs4807425	*CELF5*	19	3229224	A/G	0.268	59/266/392	0.153	0.98 (0.76–1.27)	0.903
rs9966374	*CELF4*	18	35029703	G/A	0.351	87/328/300	0.935	0.97 (0.77–1.24)	0.834
rs2495392	*CELF3*	1	151677249	A/G	0.363	95/331/291	0.936	0.95 (0.75–1.21)	0.687
rs611473	*CELF4*	18	34911038	G/A	0.108	9/135/564	0.699	1.07 (0.73–1.58)	0.722
rs1786798	*CELF4*	18	35084108	G/A	0.429	123/367/225	0.222	0.92 (0.72–1.16)	0.472
rs3740698	*CELF1*	11	47504442	A/G	0.029	0/ 42/675	1.000	1.12 (0.52–2.41)	0.766
rs1662917	*CELF4*	18	34860858	A/G	0.209	36/227/454	0.260	0.96 (0.73–1.27)	0.767
rs685680	*CELF4*	18	34905452	G/A	0.452	152/343/220	0.407	0.97 (0.77–1.22)	0.767
rs17750309	*CELF4*	18	35074761	A/G	0.478	167/350/198	0.601	1.06 (0.84–1.33)	0.643
rs1291822	*CELF2*	10	11136503	G/A	0.173	17/214/484	0.296	0.95 (0.70–1.29)	0.740
rs17452064	*CELF2*	10	11242471	C/G	0.136	12/170/532	0.873	1.04 (0.74–1.47)	0.808

(*Continued*)

SNP	Gene	Chr.	Position	Alleles[a]	MAF	GENO[b]	HWE	HR (95%CI)[c]	*P* value[c]
rs10752216	*CELF2*	10	11271928	A/G	0.161	22/187/508	0.335	1.02 (0.75–1.40)	0.887
rs9955327	*CELF4*	18	35022296	G/A	0.443	141/352/222	0.940	1.04 (0.82–1.31)	0.765
rs1786054	*CELF4*	18	34849287	G/A	0.258	46/277/392	0.845	0.98 (0.76–1.26)	0.855
rs655931	*CELF4*	18	34907964	A/G	0.331	80/314/323	0.800	1.02 (0.79–1.30)	0.902

[a]Minor allele/major allele.
[b]Minor homozygote/heterozygote/major homozygote.
[c]By COX proportional hazards regression analysis under codominant model.
MAF, minor allele frequency; HWE, Hardy-Weinberg equilibrium; HR, Hazard Ratio, adjusted for gender, age, tumor stage, and treatment; CI, confidence interval.

Supplementary Table S2: Clinical characteristics of patients with nasopharyngeal carcinoma according to rs3740194 genotypes

characteristic	Discovery (n = 717)				Validation (n = 1, 520)				Combined samples (n = 2, 237)			
	GG	AG	AA	p value*	GG	AG	AA	p value*	GG	AG	AA	p value*
**age (years)**												
< 50	27	161	145	0.299	83	367	371	0.762	110	528	516	0.870
≥ 50	34	163	186		65	323	308		99	486	494	
**Gender**												
female	13	82	96	0.346	36	180	175	0.906	49	262	271	0.583
male	48	242	235		112	510	504		160	752	739	
**T-classification**												
T1-T2	24	116	116	0.813	49	218	226	0.789	73	334	342	0.822
T3-T4	37	208	215		99	472	453		136	680	668	
**N-classification**												
N0-N1	31	181	166	0.325	74	375	372	0.563	105	556	538	0.448
N2-N3	30	143	165		74	315	307		104	458	472	
**Overall stage**												
I-II	11	70	68	0.809	24	134	137	0.544	35	204	205	0.488
III-IV	50	254	263		124	556	542		174	810	805	
**Radiotherapy**												
2D-RT	53	281	279	0.790	107	506	483	0.577	160	787	762	0.466
3D-RT	8	43	52		41	184	196		49	227	248	
**Inducing chemotherapy**												
Yes	32	177	192	0.597	74	398	390	0.145	106	575	582	0.092
No	29	147	139		74	292	289		103	439	428	
**Concurrent chemotherapy**												
Yes	40	197	200	0.747	75	350	332	0.320	115	547	532	0.258
No	21	127	131		73	340	347		94	467	478	
**Adjuvant chemotherapy**												
Yes	60	301	314	0.348	146	667	651	0.395	206	968	965	0.204
No	1	23	17		2	23	28		3	46	45	

*$\chi 2$ test by two-sided Pearson's exact test; 2D-RT, 2-dimensional radiotherapy; 3D-RT, 3-dimensional radiotherapy.

Supplementary Table S3: Primer information on the TaqMan assay of candidate SNPs in replication stage

SNP	Alleles	Primers	Probes
rs3740194	T/C	GTCTGAACACAAGCAGTG (forward)	FAM-TCCTCACTGAGCTCCTTCTCTGCACAT
		GAGAGCAAAGAGGTCCTA (reverse)	VIC-TCCTCACTGAGCTCCCTCTCTGCACAT
rs11257025	G/A	ATGGCACCTCTAGAATTG (forward)	FAM-TTGGGACTCACCAGTTGTCATT
		GGGATTACAGGGATAACC (reverse)	VIC-TTGGGACTCACCAATTGTCATT
rs7234088	C/T	GCGGTTTTATCACTAACA (forward)	FAM-CAGCTTTCTTCCTTAGCTGTGGT
		CTGCCTTCTTCATTGTAG (reverse)	VIC-CAGCTTTCTTCTTTAGCTGTGGT
rs1786814	G/A	CTGCAATCATTGCTGACTC (forward)	FAM-CCCAAGGTGGGTGAGAGGAGT
		GCTAGGCTCTGTCTTACC (reverse)	VIC-CCCAAGGTGGATGAGAGGAGT
rs7094118	C/T	CCCACATTTGTGTAAGTTG (forward)	FAM-CCTACTGAGGCCGTGAATAGAGT
		AGTCCATGGCTTGATAAG (reverse)	VIC-CCTACTGAGGCTGTGAATAGAGT

Supplementary Table S4: Association results for five candidate SNPs with NPC survival in the discovery, validation and combined stages

SNP	Gene	Alleles[a]	Stages	MAF	GENO[b]	HWE	Codominant model[c]			Dominant model[c]			Recessive model[c]		
							HR (95%CI)	*P* value	FDR[d]	HR (95%CI)	*P* value	FDR[d]	HR (95%CI)	*P* value	FDR[d]
rs3740194	*CELF2*	G/A													
			Discovery	0.312	61/324/331	0.14	0.69 (0.52–0.90)	0.007		0.67 (0.48–0.93)	0.015		0.50 (0.23–1.08)	0.071	
			Validation	0.325	148/690/679	0.16	0.70 (0.55–0.89)	0.003		0.66 (0.49–0.87)	0.004		0.61 (0.34–1.09)	0.096	
			Combined	0.321	209/ 1014/1010	0.05	0.69 (0.58–0.82)	4.16×10^{-5}	0.005	0.65 (0.53–0.81)	1.20 × 10−4	0.013	0.56 (0.35–0.88)	0.013	1.000
rs11257025	*CELF2*	A/G													
			Discovery	0.101	5/134/576	0.53	1.66 (1.18–2.33)	0.003		1.68 (1.17–2.41)	0.005		2.70 (0.66–11.1)	0.168	
			Validation	0.112	14/312/1192	0.24	1.12 (0.82–1.53)	0.470		1.19 (0.85–1.65)	0.318		0.45 (0.06–3.22)	0.426	
			Combined	0.108	19/446/1768	0.13	1.31 (1.04–1.64)	0.020	0.747	1.37 (1.07–1.75)	0.012	0.485	0.93 (0.30–2.92)	0.907	1.000
rs7094118	*CELF2*	A/G													
			Discovery	0.295	60/301/353	0.79	1.41 (1.11–1.80)	0.005		1.62 (1.16–2.27)	0.005		1.42 (0.84–2.39)	0.187	
			Validation	0.299	132/644/743	0.67	0.87 (0.69–1.09)	0.230		0.90 (0.67–1.20)	0.467		0.64 (0.35–1.18)	0.149	
			Combined	0.298	192/945/ 1096	0.58	1.08 (0.91–1.27)	0.383	1.000	1.15 (0.93–1.43)	0.194	1.000	0.94 (0.63–1.39)	0.752	1.000
rs7234088	*CELF4*	G/A													
			Discovery	0.139	8/182/525	0.08	0.64 (0.43–0.95)	0.029		0.62 (0.41–0.94)	0.025		0.71 (0.10–5.12)	0.738	
			Validation	0.123	22/329/1167	0.91	0.75 (0.54–1.04)	0.087		0.77 (0.54–1.10)	0.154		0.24 (0.03–1.70)	0.152	
			Combined	0.128	30/511/1692	0.25	0.71 (0.55–0.91)	0.008	0.448	0.71 (0.54–0.93)	0.013	0.485	0.36 (0.09–1.46)	0.153	1.000
rs1786814	*CELF4*	A/G													
			Discovery	0.086	4/115/597	0.81	0.50 (0.29–0.84)	0.009		0.50 (0.29–0.85)	0.010		0.00 (0.00-Inf)	0.995	
			Validation	0.070	7/200/1311	1.00				1.11 (0.74–1.67)	0.611		1.43 (0.20–10.3)	0.722	
			Combined	0.075	11/315/1908	0.76				0.80 (0.58–1.10)	0.170	1.000	0.72 (0.10–5.14)	0.744	1.000

[a]Minor allele/major allele. [b]Minor homozygote/heterozygote/major homozygote. [c]Codominant model: minor homozygote vs heterozygote vs major homozygote; Dominant model: minor homozygote and heterozygote vs major homozygote; Recessive model: minor homozygote vs heterozygote and major homozygote. [d]Benjamini-Hochberg correction for multiple testing. MAF, minor allele frequency; HWE, Hardy-Weinberg equilibrium; HR, Hazard Ratio, adjusted for gender, age, tumor stage, and treatment; CI, confidence interval.
